# Evaluation of Cognitive Function in Stroke Patients With Lesions in Different Brain Regions Using P300 Event-Related Potentials Combined With Video EEG

**DOI:** 10.31083/RN45402

**Published:** 2025-12-18

**Authors:** Xue Shi, Rui Zhao, Xuedong Yang, Zhuoqun Wang, Changshuai Geng, Jing Tian

**Affiliations:** ^1^Department of Neurology, Jilin Province First Automobile Works General Hospital, 130011 Changchun, Jilin, China

**Keywords:** cognitive impairment, event-related potential, P300, post-stroke cognitive impairment, stroke, video EEG, deterioro cognitivo, potencial relacionado con eventos, P300, deterioro cognitivo postictus, ictus, EEG por vídeo

## Abstract

**Objective:**

To evaluate the clinical utility of P300 event-related potentials combined with video electroencephalography (VEEG) in assessing post-stroke cognitive impairment (PSCI) in patients with strokes affecting different brain regions.

**Methods:**

Stroke patients treated at our hospital were enrolled as the observation group. Based on lesion location, stroke patients were categorized into four subgroups: frontal lobe (*n* = 59), temporal lobe (*n* = 47), basal ganglia (*n* = 73), and brainstem (*n* = 35). An additional 60 age-matched healthy individuals were recruited as controls. All participants underwent cognitive assessment using the Mini-Mental State Examination (MMSE), and P300 and VEEG evaluations.

**Results:**

At 7 days, 1 month, 3 months, and 6 months post-treatment, MMSE scores in the observation group were significantly lower than those in the control group. Correlation analysis showed that, in the frontal- and temporal-lobe groups, P300 amplitude and VEEG α and β power at day 7 were positively correlated with MMSE scores at 6 months. In contrast, P300 latency and VEEG delta and θ power, slow-wave index, and δ/α ratio (DAR) at day 7 were negatively correlated with 6-month MMSE scores. In the basal ganglia group, day 7 P300 amplitude and VEEG α power were positively correlated with 6-month MMSE scores, whereas P300 latency, δ and θ power, and DAR were negatively correlated. In the brainstem group, P300 latency, δ power, and slow-wave index at day 7 were negatively correlated with MMSE scores at 6 months. Receiver operating characteristic (ROC) analysis demonstrated that P300 combined with VEEG predicted PSCI in the frontal lobe group with a sensitivity of 94.32%, specificity of 92.58%, and area under the curve (AUC) of 0.932 (95% CI: 0.900–0.967). For the temporal lobe group, sensitivity was 82.74%, specificity 79.27%, and AUC 0.864 (95% CI: 0.812–0.915). In the basal ganglia group, sensitivity and specificity were 78.24% and 76.12%, respectively (AUC = 0.789, 95% CI: 0.727–0.851). For the brainstem group, sensitivity was 72.78%, specificity 69.56%, and AUC 0.727 (95% CI: 0.661–0.803).

**Conclusions:**

The combination of P300 and VEEG is a valuable tool for the early screening of PSCI, particularly in patients with frontal- or temporal-lobe strokes, where it shows highly predictive sensitivity and specificity.

## Abstract (es)

**Objetivo:**

Evaluar la utilidad clínica de los potenciales relacionados con eventos P300 combinados con electroencefalografía por vídeo (VEEG, *video electroencephalography*) en la evaluación del deterioro cognitivo postictus (PSCI, *poststroke cognitive impairment*) en pacientes con ictus que afectan a diferentes regiones cerebrales.

**Métodos:**

Se inscribió como grupo de observación a pacientes con accidente cerebrovascular tratados en nuestro hospital. Según la ubicación de la lesión, los pacientes con accidente cerebrovascular se clasificaron en cuatro subgrupos: lóbulo frontal (*n* = 59), lóbulo temporal (*n* = 47), ganglios basales (*n* = 73) y tronco encefálico (*n* = 35). Se incluyó a otras 60 personas sanas de la misma edad como controles. Todos los participantes se sometieron a una evaluación cognitiva mediante el Miniexamen Cognoscitivo (MMSE, *Mini-Mental State Examination*) y a evaluaciones de P300 y VEEG.

**Resultados:**

A los 7 días, 1 mes, 3 meses y 6 meses después del tratamiento, las puntuaciones del MMSE en el grupo de observación fueron considerablemente inferiores que las del grupo de control. El análisis de correlación mostró que, en los grupos de lóbulo frontal y temporal, la amplitud P300 y la potencia α y β del VEEG en el día 7 se correlacionaban positivamente con las puntuaciones del MMSE a los 6 meses. Por el contrario, la latencia P300 y la potencia delta y θ del VEEG, el índice de ondas lentas y la relación δ/α (DAR) el día 7 se correlacionaban negativamente con las puntuaciones del MMSE a los 6 meses. En el grupo de los ganglios basales, la amplitud P300 y la potencia α del VEEG en el día 7 se correlacionaron positivamente con las puntuaciones del MMSE a los 6 meses, mientras que la latencia P300, la potencia δ y θ y la DAR se correlacionaron negativamente. En el grupo del tronco encefálico, la latencia P300, la potencia δ y el índice de ondas lentas en el día 7 se correlacionaron negativamente con las puntuaciones del MMSE a los 6 meses. El análisis de la característica operativa del receptor (ROC, *receiver operating characteristic*) demostró que el P300 combinado con el VEEG predecía el PSCI en el grupo del lóbulo frontal con una sensibilidad del 94,32%, una especificidad del 92,58% y un área bajo la curva (ABC) de 0,932 (IC del 95%: 0,900–0,967). Para el grupo del lóbulo temporal, la sensibilidad fue del 82,74%, la especificidad del 79,27% y el ABC de 0,864 (IC del 95%: 0,812–0,915). En el grupo de los ganglios basales, la sensibilidad y la especificidad fueron del 78,24% y del 76,12%, respectivamente (ABC = 0,789, IC del 95%: 0,727–0,851). En el grupo del tronco encefálico, la sensibilidad fue del 72,78%, la especificidad del 69,56% y el ABC de 0,727 (IC del 95%: 0,661–0,803).

**Conclusiones:**

La combinación de P300 y VEEG es una herramienta útil para la detección precoz del PSCI, sobre todo en pacientes con accidentes cerebrovasculares en el lóbulo frontal o temporal, donde muestra una sensibilidad y especificidad altamente predictivas.

## 1. Introduction

Stroke is an acute neurological syndrome resulting from cerebral vascular injury that disrupts cerebral blood flow, typically classified as either ischemic or hemorrhagic. Globally, stroke is the second leading cause of death and a major contributor to adult disability, making it a significant public health concern [1, 2]. Although advancements in medical technology have led to decreased stroke-related mortality, the disability rate remains high. One of the most common complications is post-stroke cognitive impairment (PSCI) [3].

In China, the incidence of PSCI among patients with new-onset ischemic stroke reaches as high as 78.7% [4]. International studies have reported that the incidence of PSCI at 90 days post-stroke is approximately 71.8% [5]. A multicenter, prospective, cohort study on mild stroke patients found PSCI rates of 59% at 3 months and 51% at 18 months post-stroke, with 9% of patients exhibiting pre-existing cognitive impairment [6]. Another cohort study reported a 3-month PSCI incidence of 47.3% in patients with mild stroke [7]. Cognitive dysfunction after stroke often impairs patients’ ability to perceive and adapt to their environment, significantly affecting their activities of daily living [8]. Consequently, early prevention, diagnosis, and management of PSCI are critical for improving long-term outcomes and quality of life, and have become a focus of current research. In clinical settings, several post-stroke deficits can masquerade as cognitive impairment, notably aphasia, dysarthria, motor slowing/hemiparesis, neglect, visual loss, mood symptoms, and delirium. To distinguish aphasia from true cognitive dysfunction, brief aphasia screening plus nonverbal or picture-based tasks with alternative response formats (e.g., pointing or yes–no) help separate language output/comprehension limits from domain-level deficits. These considerations motivate the inclusion of objective, rater-independent electrophysiological measures (e.g., P300) to complement bedside scales.

PSCI in this study was defined as cognitive impairment after clinically and neuroimaging-confirmed stroke, operationalized as a Mini-Mental State Examination (MMSE) score below education-adjusted norms, with a functional impact short of dementia and exclusive of delirium or predominant language/motor/sensory confounds. A major challenge in PSCI management is the lack of objective and sensitive tools for accurately assessing cognitive impairment and its dynamic progression. Although the MMSE remains the most widely used clinical assessment tool, it primarily evaluates overt symptoms and signs [9, 10]. Its results are susceptible to interference from patients’ emotional state and from environmental factors, limiting its objectivity. Research has shown that PSCI severity is closely associated with stroke-lesion location; for instance, white-matter lesions can increase the risk of PSCI threefold, whereas cerebral atrophy and white-matter damage are associated with a 2–3-fold increase in the risk of post-stroke dementia [11]. However, MMSE lacks the ability to localize lesions or track cognitive processing in real time. Therefore, identifying reliable and objective methods capable of dynamically assessing cognitive function and localizing PSCI-related lesions is essential for precise diagnosis and prognosis.

Examination of event-related potentials (ERPs) offers a noninvasive electrophysiological approach to evaluating brain cognitive function. Among ERP characteristics, the P300 component is the most widely used. Emerging approximately 300 ms after stimulus onset, P300 is linked to activity across multiple brain regions and is considered a core component in cognitive neuroscience research [12]. The P300 latency reflects the time required for stimulus evaluation, and its amplitude indicates the allocation of attentional resources and cognitive control [13]. In patients with PSCI, P300 latency is significantly prolonged and amplitude reduced, suggesting impaired neural processing speed and reduced recruitment of functional neurons during cognitive tasks [14]. Notably, P300 is particularly sensitive to deficits in attention and executive control, with secondary relevance to working memory; it has been applied to screen or track cognitive impairment after stroke and in prodromal neurodegenerative conditions in which attentional or executive dysfunction is prominent [15, 16, 17]. Compared with conventional bedside scales, for example, MMSE and Montreal Cognitive Assessment (MoCA) P300 provides rater-independent, quantitative indices (latency and amplitude) that can capture subtle electrophysiological alterations potentially preceding overt behavioral changes, thereby complementing scale-based assessments and facilitating objective longitudinal monitoring [18, 19].

Electroencephalography (EEG) is another noninvasive tool that is widely accepted for studying brain electrophysiological activity, and is also applicable in PSCI assessment [20]. Although conventional EEG has limitations in spatial resolution and recording duration, video EEG (VEEG) offers continuous 24-h monitoring and captures clinical symptoms, providing superior diagnostic accuracy [21, 22]. Although numerous studies have utilized MMSE and P300 to assess cognitive function in stroke patients, research combining these tools with EEG to evaluate cognitive impairment in relation to stroke lesion location remains limited.

The present study investigated the dynamic changes in cognitive function among stroke patients with different lesion locations, using MMSE, P300, and VEEG assessments. By analyzing regional differences in PSCI presentation and predicting the long-term risk of cognitive impairment, we seek to guide early interventions tailored to lesion location. The ultimate goal is to prevent or delay the onset of severe PSCI and improve the long-term prognosis and quality of life in stroke survivors.

## 2. Methods

### 2.1 Participants

A total of 214 patients diagnosed with stroke and treated at our hospital from January to December 2024 were enrolled as the observation group. To address potential region-specific differences in cognitive circuitry, patients were grouped by primary lesion location with the *a priori* purpose of comparing whether the prognostic utility of P300 and VEEG for PSCI differed across neuroanatomically distinct regions (frontal lobe, temporal lobe, basal ganglia, brainstem). Grouping information was prespecified for use in region-wise baseline comparisons, correlation analyses between day 7 electrophysiology and 6-month MMSE, and region receiver operating characteristic (ROC) analyses for predicting PSCI. All patients were treated with guideline-directed standard care consistent with American Heart Association (AHA)/American Stroke Association (ASA) recommendations and no investigational therapies were assigned within this study [23].

### 2.2 Inclusion Criteria

The inclusion criteria were: (1) diagnosis met the criteria established at the Fourth National Conference on Cerebrovascular Diseases, and stroke was confirmed by cranial CT or MRI [24]; (2) age between 50 and 70 years; (3) single-lesion stroke with the responsible lesion located in a defined brain region; (4) first-ever stroke with admission within 24 h of onset; (5) stable vital signs and neurological symptoms for over 24 h with clear consciousness; (6) informed consent obtained from patients and their families, with ethical approval from the hospital’s Ethics Committee.

### 2.3 Exclusion Criteria

Exclusion criteria were: (1) history of traumatic brain injury, previous stroke, brain tumor, epilepsy, Parkinson’s disease, encephalitis, meningitis, Alzheimer’s disease, multiple system atrophy, frontotemporal dementia, or motor neuron disease; (2) pre-existing cognitive impairment; (3) severe aphasia or visual/auditory deficits precluding cognitive testing; (4) comorbid anxiety, depression, or psychiatric disorders; (5) cognitive decline due to non-vascular causes; (6) history of alcohol/drug dependence or sleep disorders; (7) recent use of psychiatric medications or drugs known to affect cognition or EEG results. An age-matched control group of 60 healthy individuals was recruited during routine physical examinations. Controls had no history of stroke, self-reported no cognitive decline, and had MMSE scores ≥27. The same exclusion criteria applied.

### 2.4 Observation Group

Among the observation group, there were 143 males and 71 females, aged 50–70 (62.47 ± 3.28) years. Body mass index (BMI) ranged from 20.44 to 27.43 (25.13 ± 2.83). Educational level: 53 patients had a junior high school education or below, 125 had completed high school or vocational training, and 36 had college-level education or higher. The control group included 41 males and 19 females with similar BMI and educational backgrounds. There were no significant differences between groups in age, sex, BMI, or education level (*p* > 0.05). Patients in the observation group were further categorized based on lesion location: frontal lobe (*n* = 59), temporal lobe (*n* = 47), basal ganglia (*n* = 73), or brainstem (*n* = 35).

### 2.5 Neuroimaging

All patients in the observation group underwent cranial CT or MRI within 2 days of symptom onset. Imaging was independently reviewed by two board-certified neuroradiologists who were blind to MMSE and electrophysiology results. The primary (dominant) acute lesion was defined by concordance of imaging extent and clinical syndrome; when needed, dominance was adjudicated by the largest acute lesion volume and the strongest symptom–lesion correspondence. Based on the dominant lesion, each patient was assigned to one of four regions: frontal lobe, temporal lobe, basal ganglia, or brainstem. Patients with multifocal acute lesions spanning two or more of these regions without a clearly dominant site were excluded from region-wise comparison and ROC analyses (but retained in overall descriptive statistics, if applicable). Disagreements were resolved by a third senior neuroradiologist; inter-rater agreement was recorded. Lesion type (ischemic or hemorrhagic) was documented for descriptive analyses.

### 2.6 MMSE Assessment

All MMSE assessments were conducted by trained personnel in a quiet and distraction-free environment. In both the observation and control groups, MMSE was assessed at prespecified follow-up visits (7 days, 1 month, 3 months, and 6 months) according to a uniform schedule. The reported between-group comparisons at each visit reflected average effects across potentially heterogeneous stroke mechanisms. Patients were informed of the assessment purpose, and items were administered in sequence using standardized instructions. Responses were recorded immediately. If any item could not be completed due to external factors, the reason was documented and testing continued. Total scores were verified on-site and entered into the database within 24 h, followed by double-checking to ensure accuracy. The total MMSE score is 30, with higher scores indicating better cognitive function. Scores were adjusted for educational level: individuals with ≤4 years of education received a 2-point adjustment, and those with 4–8 years received a 1-point adjustment. A score ≥27 was considered normal cognition.

### 2.7 P300 Event-Related Potential (ERP) Recording

Before ERP, all participants underwent otoscopy, pure-tone audiometry (0.5–4 kHz; excluded if better-ear Pure Tone Average (PTA) >25 dB Hearing Level (HL)), and click-evoked Brainstem Auditory Evoked Potential (BAEP) at 70–80 dB nHL with exclusion when absolute wave latencies or I–V interpeak intervals exceeded laboratory norms. P300 was recorded with Cz referenced to linked mastoids and Electro-oculography (EOG) monitoring, using an auditory oddball with 1000 Hz standards and 2000 Hz targets (80/20), intensity 70–75 dB Sound Pressure Level (SPL), Inter-Stimulus Interval (ISI) 1000 ms (±10% jitter), and 300 trials. Data were sampled at 1000 Hz, bandpass filtered 0.1–30 Hz, epoched –100 to 800 ms, with artifact rejection at ±75 µV. The P300 at Cz was defined as the maximal positive deflection at 250–450 ms; latency was measured to peak and amplitude as peak-to-baseline. Task comprehension was verified by a 20-trial practice requiring ≥80% correct.

P300 was recorded at 9:00 AM in a sound- and light-shielded room using the Danish Dantec Keypoint 9033A07 evoked potential system (Dantec, Skovlunde, Denmark). Patients removed all metallic items, and the scalp was cleaned to reduce impedance. Electrodes were applied using conductive paste. Subjects wore headphones, maintained a relaxed and alert state, and underwent an auditory-brainstem-response test to exclude hearing deficits. Electrodes were placed at Frontal zero (Fz), Central zero (Cz), and Parietal zero (Pz) according to the international 10–20 system; reference electrodes were placed on the mastoids and the ground electrode at the mid-forehead. Impedance was kept below 5 kΩ. Stimuli followed an auditory Oddball paradigm: target tones (90 dB, 20% of stimuli) and non-target tones (60 dB, 80%) were presented randomly. Subjects responded to target tones by pressing a button. Thirty trials were averaged with a recording window of 1000 ms, sensitivity 10 µV/division (div), and bandpass filter of 10 Hz to 3 kHz. P300 latency and amplitude were recorded at Cz, in accordance with international ERP standards. P300 indices were analyzed both overall and stratified by lesion-location groups.

### 2.8 VEEG Monitoring

VEEG preprocessing and spectral analysis used EEGLAB (v14.1.1, Swartz Center for Computational Neuroscience, San Diego, CA, USA) and MATLAB (R2017a, Mathworks, Natick, MA, USA). Signals were rereferenced to common average, bandpass filtered 0.5–45 Hz with 50/60 Hz notch, downsampled to 256 Hz, and cleaned via Independent Component Analysis (ICA) (removal of ocular/muscle components). Artifactfree data were epoched into 2s windows (50% overlap). Power spectral density was estimated by Welch’s method (Hamming, 2s, 50% overlap), and absolute band powers were integrated for alpha (α), beta (β), delta (δ), and theta (θ); slowwave index was (δ + θ)/(α + β); DAR was δ/α. We analyzed P300 amplitude and latency alongside VEEG metrics, summarizing globally across electrodes. Lesionlocation stratification (frontal, temporal, basal ganglia, brainstem) was prespecified; controls were not stratified. A minimum of 20-min artifactfree EEG was required.

Continuous 24-h EEG monitoring was performed using a digital EEG recording system (XE-W-36-S, Swartz Center for Computational Neuroscience). Electrode placement followed the international 10–20 system, including 16 recording electrodes and 2 references. Sampling rate was 512 Hz with a filter range of 0.1–100 Hz. VEEG signals were analyzed using EEGLAB (v14.1.1, Swartz Center for Computational Neuroscience), Edit 4.5 (Swartz Center for Computational Neuroscience), and in-house software (R8.1, Swartz Center for Computational Neuroscience). Absolute power of α, β, δ, and θ bands was computed. The slow-wave index was calculated as (δ + θ)/(α + β), and the δ/α ratio (DAR) was derived.

### 2.9 Quality Control

To reduce selection bias, strict adherence to inclusion and exclusion criteria was maintained. Baseline characteristics were compared to control for confounding. All assessors were trained in standardized protocols, and communication with patients was consistent. Before testing, participants were asked to rest, eat, and ensure scalp cleanliness. Metal objects and electronic devices were removed. During tests, participants were instructed to stay alert and avoid excessive blinking. Data entry was performed by one person and verified by two individuals to minimize input errors before statistical analysis.

### 2.10 Statistical Analysis

Statistical analysis was conducted using SPSS version 22.0 (IBM Corp., Armonk, NY, USA). The Kolmogorov–Smirnov test was used to assess the normality of continuous variables. Data conforming to a normal distribution were expressed as mean (SD). Comparisons between two groups were performed using the *t*-test, and comparisons among multiple groups used Analysis of Variance (ANOVA). Pearson correlation was used to evaluate associations between MMSE scores and electrophysiological parameters. Before computing Pearson correlations within each lesion group, we assessed bivariate normality and linearity using univariate normality tests (K–S/Shapiro–Wilk), scatterplots with ellipse-based diagnostics and Mahalanobis distance, and residual plots. For skewed ratio-type metrics (e.g., DAR, slow-wave index), log/Box–Cox transformations were applied; when assumptions remained violated or *n* was small, Spearman’s rho was used as the primary estimate with Pearson reported as supportive, and multiple testing was controlled using Benjamini-Hochberg False Discovery Rate (BH-FDR) within groups. ROC analysis was used to assess the predictive value of different indicators for PSCI. A *p*-value ≤ 0.05 was considered statistically significant. Pairwise comparisons of AUC values across lesion groups were performed using DeLong tests, with Bonferroni correction applied for multiple comparisons (α = 0.05/6 ≈ 0.0083).

## 3. Results

### 3.1 Comparison of MMSE Scores Between Observation and Control Groups

Baseline characteristics (age, sex, BMI, education) were comparable between groups (all *p* > 0.05). Detailed values are provided in **Supplementary Table 1**. At 7 days, 1 month, 3 months, and 6 months after treatment, MMSE scores in the observation group remained significantly lower than those in the control group (*p* < 0.05). Detailed results are presented in Table 1.

**Table 1.** **Comparison of MMSE scores between observation and control groups at different time points post-treatment**.

Group	*n*	7 days	1 month	3 months	6 months
Observation group	214	21.87 ± 2.18	24.45 ± 2.27	25.14 ± 2.22	26.53 ± 2.51
Control group	60	28.75 ± 0.72	28.75 ± 0.72	28.75 ± 0.72	28.75 ± 0.72
t		24.053	14.454	12.400	6.765
*p*		<0.001	<0.001	<0.001	<0.001

### 3.2 Trends in MMSE Scores Among Stroke Patients With Lesions in Different Brain Regions

All stroke subgroups showed significantly lower MMSE scores than did control group at 7 days and 1 month post-treatment (*p* < 0.05). At 3 and 6 months, MMSE scores in the frontal lobe, temporal lobe, and basal ganglia groups remained significantly lower than control scores (*p* < 0.05). At 7 days and 1 month, the frontal and temporal lobe groups had significantly lower MMSE scores than did the basal ganglia and brainstem groups (*p* < 0.05). At 3 months, MMSE scores in the frontal lobe group were lower than those in the temporal lobe and basal ganglia groups, and the temporal lobe and basal ganglia groups scored lower than the brainstem group (*p* < 0.05). At 6 months, a progressive trend was observed: MMSE scores were lowest in the frontal lobe group, followed by the temporal lobe group, then the basal ganglia group, with the brainstem group scoring highest (*p* < 0.05). Detailed comparisons are shown in Table 2.

**Table 2.** **Longitudinal comparison of MMSE scores among stroke patients with lesions in different brain regions**.

Group	*n*	7 days	1 month	3 months	6 months
Frontal lobe	59	18.47 ± 2.13^acd^	20.25 ± 2.32^acd^	21.24 ± 2.21^abd^	23.15 ± 2.34^abd^
Temporal lobe	47	19.34 ± 1.98^acd^	20.91 ± 2.18^acd^	24.94 ± 2.27^ad^	25.09 ± 2.48^ac^
Basal ganglia	73	22.45 ± 2.21^a^	24.84 ± 2.29^a^	25.78 ± 2.23^ad^	26.31 ± 2.02^ad^
Brainstem	35	22.89 ± 2.06^a^	25.93 ± 2.30^a^	27.97 ± 2.94^a^	28.28 ± 2.79
Control	60	28.75 ± 0.72	28.75 ± 0.72	28.75 ± 0.72	28.75 ± 0.72
F		15.824	13.306	12.274	10.724
*p*		<0.001	<0.001	<0.001	<0.001

^a^*p* < 0.05 *vs* control group; ^b^*p* < 0.05 *vs* temporal lobe group; ^c^*p* < 0.05 *vs* basal ganglia group; ^d^*p* < 0.05 *vs* brainstem group.

### 3.3 Comparison of P300 Parameters at Day 7 Among Stroke Patients With Different Lesion Locations

At 7 days post-stroke, all lesion groups exhibited significantly reduced P300 amplitudes and prolonged latencies than did control group (*p* < 0.05). Among the stroke subgroups, the frontal and temporal lobe groups showed the lowest P300 amplitudes. The basal ganglia group exhibited intermediate values, and the brainstem group had relatively preserved amplitudes (*p* < 0.05). For P300 latency, the frontal lobe group exhibited the most prolonged values, followed by the temporal lobe group, both of which were significantly longer than those in the basal ganglia and brainstem groups (*p* < 0.05). Detailed comparisons are presented in Table 3.

**Table 3.** **Comparison of P300 amplitude and latency among stroke patients with different lesion locations at day 7**.

Group	*n*	Amplitude	Latency
Frontal lobe	59	1.62 ± 1.33^acd^	482.94 ± 19.73^abcd^
Temporal lobe	47	1.93 ± 1.21^acd^	443.82 ± 21.35^acd^
Basal ganglia	73	2.97 ± 1.28^ad^	384.74 ± 14.28^a^
Brainstem	35	4.15 ± 1.61^a^	372.27 ± 23.48^a^
Control	60	5.12 ± 1.23	342.84 ± 52.83
F		25.824	19.952
*p*		<0.001	<0.001

^a^*p* < 0.05 *vs* control group; ^b^*p* < 0.05 *vs* temporal lobe group; ^c^*p* < 0.05 *vs* basal ganglia group; ^d^*p* < 0.05 *vs* brainstem group.

### 3.4 Comparison of VEEG Parameters at Day 7 Among Stroke Patients With Different Lesion Locations

At 7 days post-stroke, VEEG parameters showed significant differences between lesion groups and healthy controls. In the frontal and temporal lobe groups, α and β wave power were significantly reduced, while δ and θ wave power, slow-wave index, and DAR were significantly elevated (*p* < 0.05). In the basal ganglia group, α wave power was markedly decreased, with increased δ and θ power and DAR (*p* < 0.05). In the brainstem group, only δ wave power and the slow-wave index were significantly elevated compared with controls (*p* < 0.05). Further comparisons revealed that the frontal lobe group had lower α power and higher DAR than the temporal lobe group (*p* < 0.05). Both the frontal and temporal lobe groups showed lower α and β power, and higher δ, θ power, slow-wave index, and DAR than did the basal ganglia and brainstem groups (*p* < 0.05). Additionally, the basal ganglia group had higher DAR than the brainstem group (*p* < 0.05). Detailed values are presented in Table 4.

**Table 4.** **Comparison of VEEG parameters at day 7 among stroke patients with different lesion locations**.

Group	*n*	α wave power (μV^2^)	β wave power (μV^2^)	δ wave power (μV^2^)	θ wave power (μV^2^)	Slow wave index	DAR
Frontal lobe	59	3.23 ± 1.03^abcd^	2.89 ± 1.19^acd^	33.82 ± 16.38^acd^	32.72 ± 9.13^acd^	11.18 ± 3.14^acd^	10.36 ± 3.55^abcd^
Temporal lobe	47	4.18 ± 1.24^acd^	2.57 ± 1.42^acd^	32.63 ± 13.27^acd^	28.89 ± 8.79^acd^	9.89 ± 3.21^acd^	7.89 ± 3.79^acd^
Basal ganglia	73	6.24 ± 2.17^a^	6.63 ± 1.93	26.85 ± 11.32^a^	21.73 ± 9.29^a^	3.66 ± 2.48	4.32 ± 2.69^ad^
Brainstem	35	7.12 ± 2.39	5.89 ± 1.48	21.54 ± 7.29^a^	14.48 ± 8.73	3.24 ± 1.87^a^	2.13 ± 1.98
Control	60	7.97 ± 2.89	6.24 ± 1.21	15.82 ± 6.27	13.96 ± 5.24	1.45 ± 0.89	0.78 ± 0.63
F		14.922	18.846	19.842	13.296	21.982	19.829
*p*		<0.001	<0.001	<0.001	<0.001	<0.001	<0.001

^a^*p* < 0.05 *vs* control group; ^b^*p* < 0.05 *vs* temporal lobe group; ^c^*p* < 0.05 *vs* basal ganglia group; ^d^*p* < 0.05 *vs* brainstem group.

### 3.5 Correlation Between Day 7 P300 and VEEG Parameters and MMSE Scores at 6 Months in Stroke Patients

Correlation analyses revealed that, in the frontal and temporal lobe groups, P300 amplitude and VEEG α and β wave power measured at 7 days post-treatment were positively correlated with MMSE scores at 6 months (*p* < 0.001). In contrast, P300 latency, VEEG δ and θ wave power, slow-wave index, and DAR were negatively correlated with MMSE scores at 6 months (*p* < 0.001). In the basal ganglia group, both P300 amplitude and α wave power were positively correlated with 6-mo MMSE scores, whereas P300 latency, δ and θ wave power, and DAR showed negative correlations (*p* < 0.05). In the brainstem group, P300 latency, δ wave power, and the slow-wave index were negatively correlated with MMSE scores at 6 s (*p* < 0.05). Correlation coefficients and significance levels are shown in Table 5.

**Table 5.** **Correlation between P300 and VEEG parameters at day 7 and MMSE scores at 6 months in stroke patients by lesion location**.

Group	P300 amplitude	P300 latency	α wave power	β wave power	δ wave power	θ wave power	Slow wave index	DAR
Frontal lobe	0.723**	–0.711**	0.734**	0.621**	–0.482**	–0.367**	–0.763**	–0.755**
Temporal lobe	0.683**	–0.608**	0.528**	0.589**	–0.469**	–0.348**	–0.634**	–0.647**
Basal ganglia	0.392**	–0.383*	0.407*	0.104	–0.339*	–0.354*	–0.118	–0.363*
Brainstem	0.137	–0.316*	0.121	0.089	–0.312*	–0.093	–0.325*	–0.110

*p < 0.05; **p < 0.001.

### 3.6 ROC Analysis of P300 Combined With VEEG for Predicting PSCI in Different Stroke-Lesion Locations

**Fig. 1.**
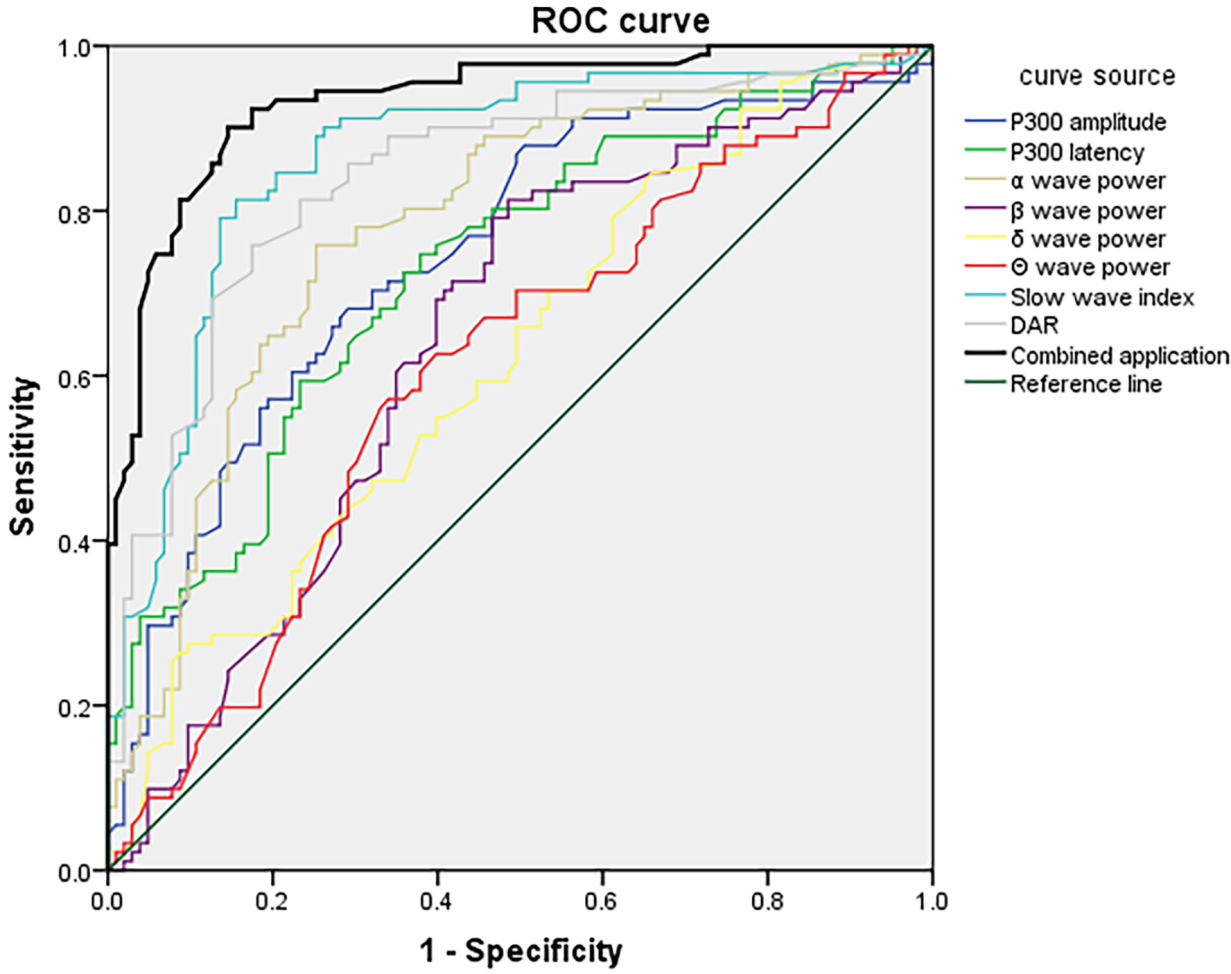
**ROC curve of P300 combined with VEEG parameters at day 7 for predicting PSCI in patients with frontal lobe infarction**. ROC, receiver operating characteristic; VEEG, video electroencephalography; PSCI, post-stroke cognitive impairment; DAR, δ/α ratio.

**Fig. 2.**
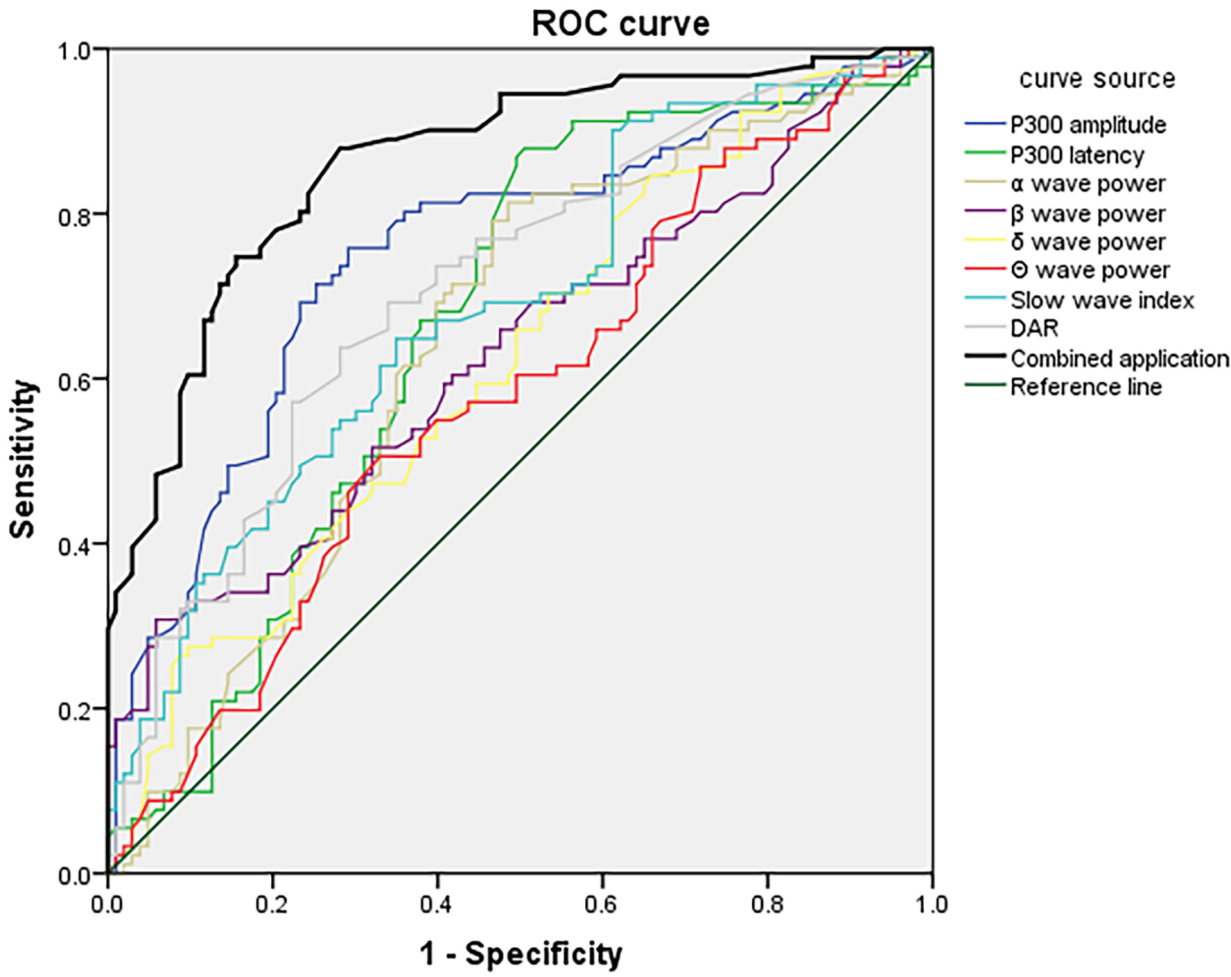
**ROC curve of P300 combined with VEEG parameters at day 7 for predicting PSCI in patients with temporal lobe infarction**.

**Fig. 3.**
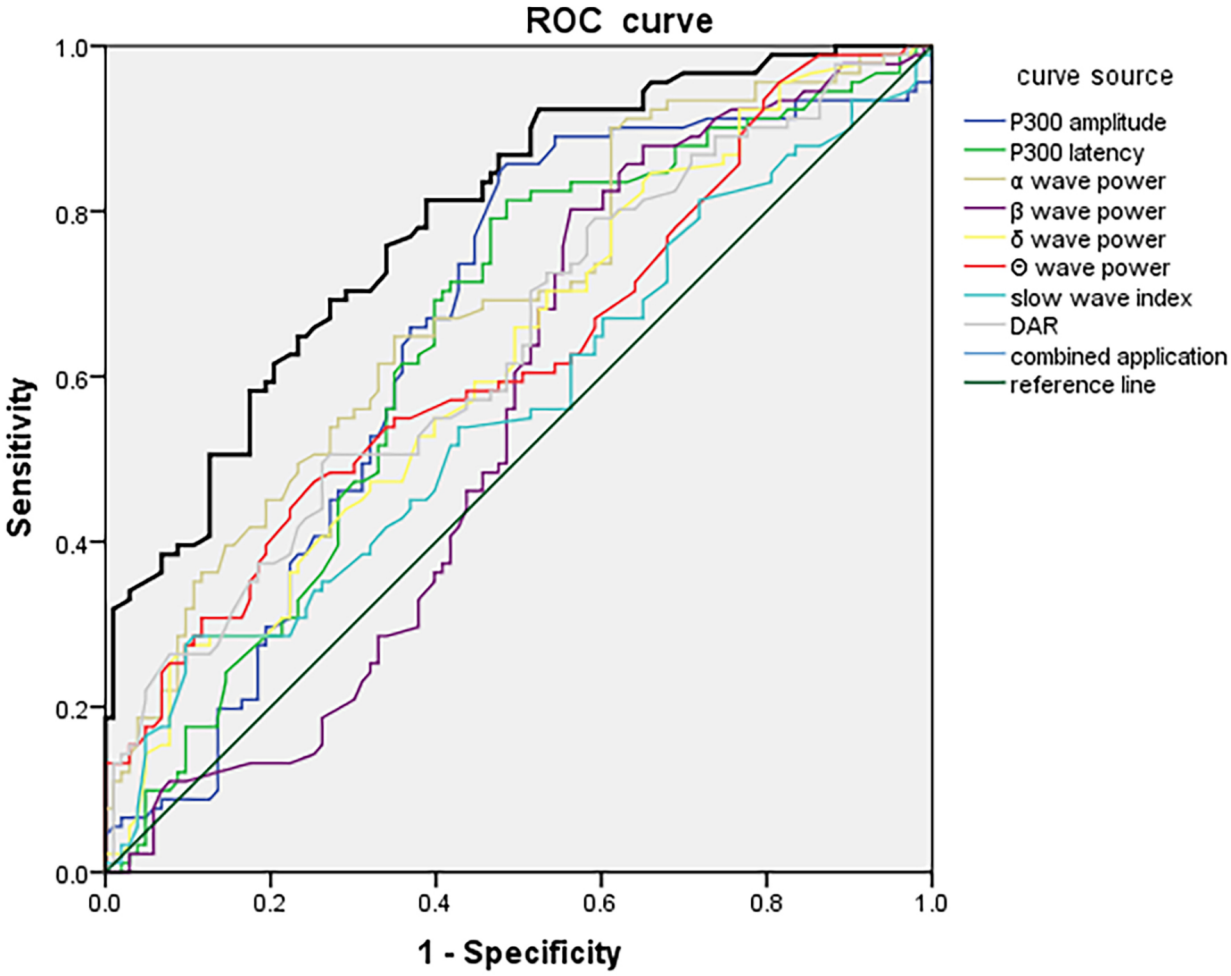
**ROC curve of P300 combined with VEEG parameters at day 7 for predicting PSCI in patients with basal ganglia infarction**.

**Fig. 4.**
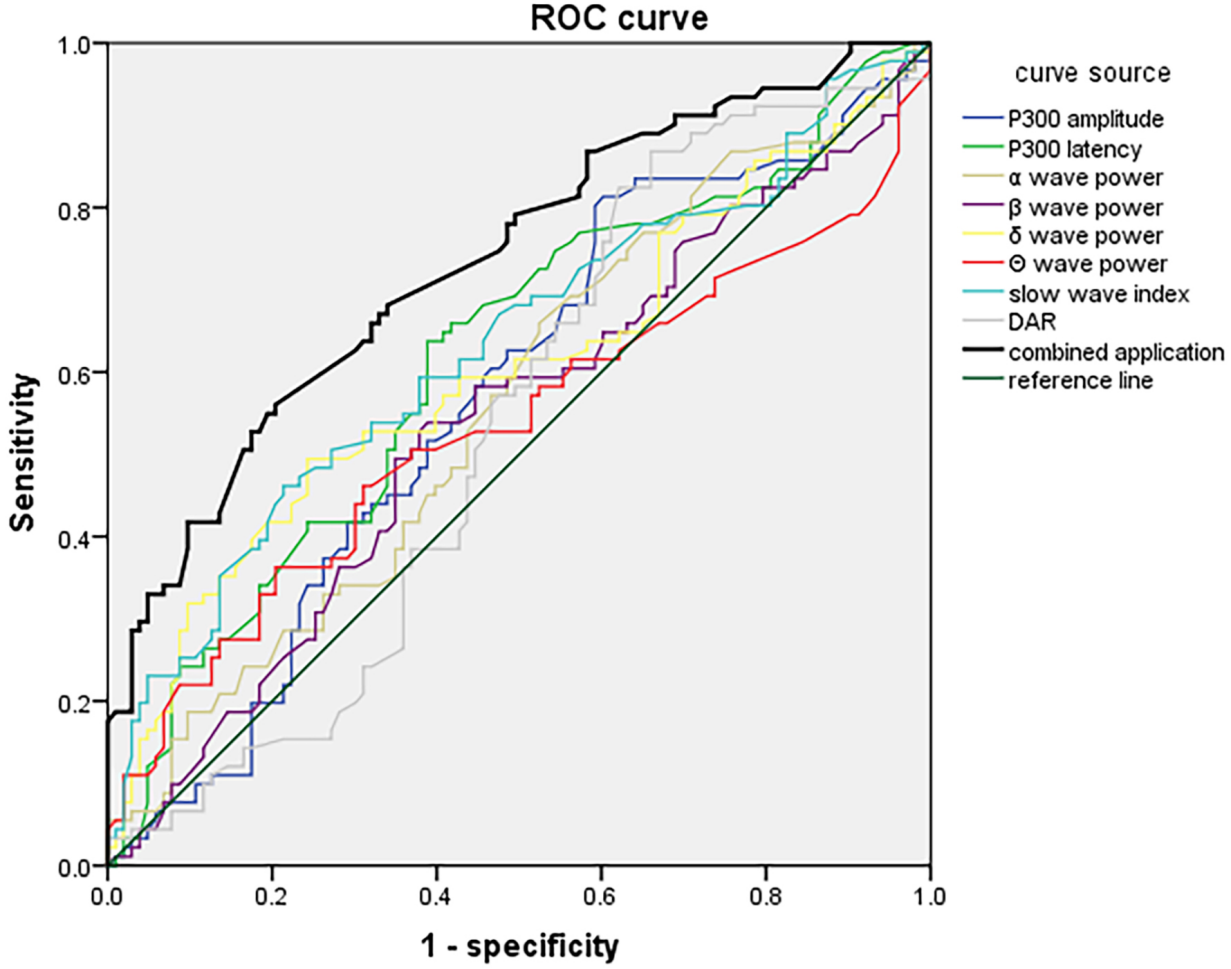
**ROC curve of P300 combined with VEEG parameters at day 7 for predicting PSCI in patients with brainstem infarction**.

ROC curves were constructed using P300 latency and amplitude combined with VEEG parameters (α, β, δ, θ wave power, slow-wave index, and DAR) as test variables. The presence or absence of PSCI in each lesion group was used as the outcome variable. In the frontal lobe group, the combined P300 and VEEG model yielded a sensitivity of 94.32% and a specificity of 92.58%, with an AUC of 0.932 (95% CI: 0.900–0.967), indicating excellent predictive performance (Fig. 1). In the temporal lobe group, the combined model achieved a sensitivity of 82.74%, specificity of 79.27%, and an AUC of 0.864 (95% CI: 0.812–0.915), demonstrating good predictive ability (Fig. 2). For the basal ganglia group, the model showed a sensitivity of 78.24%, specificity of 76.12%, and an AUC of 0.789 (95% CI: 0.727–0.851), indicating moderate diagnostic accuracy (Fig. 3). In the brainstem group, the combined model demonstrated a sensitivity of 72.78%, specificity of 69.56%, and an AUC of 0.727 (95% CI: 0.661–0.803), suggesting fair predictive value (Fig. 4). These results indicated that P300 combined with VEEG had the highest predictive accuracy for PSCI in patients with frontal lobe lesions, followed by those with temporal, basal ganglia, and brainstem lesions. Pairwise DeLong tests with Bonferroni correction revealed that the frontal lobe model significantly outperformed the basal ganglia (*p* = 0.002) and brainstem models (*p* < 0.001). The temporal lobe model also significantly exceeded the brainstem model (*p* = 0.006). The difference between frontal and temporal models was not significant after correction (*p* = 0.018).

## 4. Discussion

This study found that the MMSE scores of stroke patients were significantly lower than those of the control group within 7 days to 6 months, and there were significant differences in the recovery trajectories at different locations. Lesions in the frontal and temporal lobes showed more severe and persistent P300 impairment, α inhibition, and slow-wave enhancement, the basal ganglia exhibited rhythm imbalance, and the brainstem showed the mildest changes. At 7 days, the prolonged P300 latency and decreased amplitude, as well as the predominance of slow waves in VEEG and the increase in DAR, were significantly correlated with the MMSE scores at 6 months. The P300 combined with the VEEG model was superior to a single indicator in predicting PSCI in the frontal and temporal lobes and achieved higher AUC, sensitivity, and specificity.

Previous guidelines and systematic reviews have indicated that although the MMSE is convenient for rapid screening, it has insufficient sensitivity in executive and visuospatial domains. Beyond bedside scales, early electrophysiological markers offer an objective, domain-agnostic assessment that is less confounded by language and education. Prior meta-analyses recommend MoCA for mild PSCI, whereas ERP and EEG provide complementary physiological sensitivity to executive and attentional dysfunction. Therefore, identifying objective neurophysiological tools for early PSCI detection is crucial. Event-related potential P300 and VEEG offer high temporal resolution, non-invasiveness, and suitability for continuous monitoring. This study investigated the association between P300/VEEG parameters and MMSE scores in stroke patients with lesions in different brain regions and evaluated their predictive accuracy for long-term PSCI risk.

Consistent with previous studies [3, 4, 5, 6, 7], MMSE scores in stroke patients were significantly lower than those in healthy controls at all post-treatment times, underscoring the acute and persistent detrimental effects of stroke on cognitive function [3, 4, 5, 6, 7]. This decline may be attributed to ischemia- and hypoxia-induced pathological changes, such as neuronal necrosis, axonal demyelination, and structural damage [25, 26, 27], compounded by secondary injuries including cerebral edema and inflammatory response [28, 29]. This pattern underscores the rationale for early risk stratification and targeted cognitive rehabilitation.

Cognitive recovery trajectories were closely linked to lesion location. Frontal lobe infarction patients showed persistently low MMSE scores with minimal improvement, indicative of damage to higher-order cognitive control centers. The temporal lobe group exhibited significant early decline, particularly in language and memory, with partial recovery after 3 months likely due to neurofunctional restoration and compensatory reorganization within the temporal-limbic network [25]. Basal ganglia lesions led to relatively milder declines, though deficits in processing speed and complex task execution persisted. In contrast, the brainstem group experienced only transient cognitive impairment, with rapid recovery as brainstem function stabilized, suggesting less direct involvement in cognitive processing. These lesion-specific trajectories aligned with reports that frontal strokes yield the most persistent executive deficits, temporal strokes predominantly affect language and memory with partial recovery, whereas basal ganglia and brainstem lesions often show greater potential for network-level compensation.

As a non-invasive electrophysiological marker, P300 latency reflects the speed of information processing, whereas amplitude indicates cognitive resource allocation [30]. In this study, P300 alterations at 7 days post-stroke exhibited distinct lesion-specific patterns. Frontal lobe infarction was associated with the most pronounced prolongation of latency and reduction in amplitude, indicating substantial impairment of the prefrontal cognitive network. The temporal lobe group showed comparable amplitude reduction but less prolonged latency, suggesting suppressed efficiency of the auditory cortex–hippocampal pathway [31]. The basal ganglia group exhibited mild latency prolongation but marked amplitude instability, possibly reflecting impaired thalamocortical regulation, whereas the brainstem group showed only subtle P300 changes, likely due to disrupted arousal regulation [32]. These findings suggest that the severity of P300 abnormalities varies by lesion location and may correspond to differential risk for PSCI. Specifically, early and marked P300 impairment, characterized by prolonged latency and attenuated amplitude, is more pronounced in frontal and temporal lobe strokes, indicating a higher likelihood of developing post-stroke cognitive dysfunction in these subgroups.

Correlation analysis revealed that in the frontal, temporal, and basal ganglia groups, P300 amplitude at 7 days post-stroke was positively associated with MMSE scores at 6 months, whereas P300 latency showed a negative correlation. In the brainstem group, only P300 latency was negatively correlated with 6-month MMSE scores. These findings indicated that early changes in P300 parameters, particularly amplitude and latency, may have predictive value for long-term cognitive outcomes. Among all lesion locations, the frontal lobe group exhibited the strongest correlation between early P300 abnormalities and subsequent cognitive impairment. This is consistent with the role of the frontal cortex in high-order cognitive functions such as memory, judgment, and executive control. Frontal stroke often leads to widespread and persistent cognitive deficits, and early reductions in P300 amplitude and prolonged latency may indicate enduring impairments in cognitive task processing at 6 months. In the temporal lobe group, early P300 parameters showed moderate correlation with 6-month MMSE scores, suggesting an elevated risk of long-term deficits in language and episodic memory. However, partial recovery may occur due to compensatory reorganization within the temporal–limbic system [33]. In the basal ganglia group, early P300 indices were also moderately correlated with long-term MMSE scores. This may reflect gradual cognitive decline after disruption of thalamocortical projection pathways. Nevertheless, some patients in this group demonstrated trends toward MMSE score improvement, indicating potential for functional compensation. In the brainstem group, P300 latency at day 7 was weakly but significantly correlated with cognitive performance at 6 months.

Since brainstem damage primarily affects the ascending reticular activating system, the early latency abnormalities likely reflected transient impairments in alertness. As brainstem function recovers, the overall risk of persistent PSCI is relatively low, though subtle deficits in attention and vigilance may persist. These findings suggest that frontal and temporal lobe strokes produce more pronounced and sustained disruptions in P300 components, which are less amenable to compensation, whereas lesions in the basal ganglia and brainstem may allow for partial neurofunctional recovery through network reorganization. The observed region-specific relationships between early P300 alterations and long-term cognitive outcomes provide an objective electrophysiological basis for stratifying PSCI risk in stroke patients. This is in line with findings by Sheema and Rawekar [34], who reported that although P300 is a useful biomarker for predicting cognitive impairment, its predictive power is limited when used alone and should be integrated with other neuroimaging modalities for more comprehensive and dynamic assessment.

The results of this study demonstrated varying degrees of VEEG abnormalities in stroke patients across different lesion locations at 7 days post-onset. Previous research has shown that EEG is a sensitive tool for detecting cerebral hypoperfusion, where suppressed cerebral blood flow is typically associated with decreased α and β band power and increased θ-band activity [35]. Our findings were consistent with these observations. Additionally, previous studies have reported that quantitative EEG (qEEG) parameters, particularly the DAR, correlate with infarct volume. A reduction in DAR to sub-threshold levels has been associated with successful reperfusion therapy, whereas persistently elevated DAR values indicate treatment failure. These findings are in agreement with the results of the present study [36]. Moreover, sub-threshold normalization of DAR after reperfusion has been linked to functional recovery, supporting DAR as a dynamic biomarker for monitoring treatment response.

Further analysis of VEEG parameters across stroke locations revealed distinct electrophysiological patterns. The frontal lobe group exhibited a marked reduction in α-wave power accompanied by significant increases in δ wave and θ wave power, slow-wave index, and DAR. These findings indicated suppressed cortical excitability and enhanced slow-wave activity, reflecting severe disruption of high-level cognitive control networks. In the temporal lobe group, the decline in α wave power was less pronounced than in the frontal group, yet the elevation in θ wave power and DAR was comparable. Additionally, the slow-wave index was significantly higher than in the basal ganglia and brainstem groups, suggesting that temporal lobe lesions impair the hippocampal–medial temporal memory encoding regions. This aligned with the findings of Bailey *et al*. [37], who reported that abnormal triphasic wave energy during stroke is predominantly localized to frontal and temporal electrodes. In the present study, the basal ganglia group showed a mild decrease in α wave power and a slight increase in β wave power, potentially reflecting compensatory excitability adjustments within cortical neurons under pathological conditions. Concurrent abnormalities in δ wave, θ wave, and DAR point to disrupted cortical–basal ganglia rhythmic regulation, dominated by an imbalance between slow and fast waves. Previous studies have indicated that α-wave-power reduction coupled with β-wave-power elevation may represent a compensatory neuronal excitation in response to hypoxia during early brain injury or chronic ischemic damage. Although the brainstem group demonstrated the least overall EEG power alteration, prominent abnormalities were observed in δ-wave power and slow-wave index, suggesting injury to the ascending reticular activating system and resultant impairment in arousal maintenance.

Correlation analysis revealed varying degrees of association between VEEG parameters at 7 days post-stroke and MMSE scores at 6 months. Patients with frontal and temporal lobe strokes exhibited a “slow wave dominant” pattern characterized by α-wave suppression alongside marked increases in δ- and θ-wave power. These VEEG abnormalities showed moderate to strong correlations with MMSE scores at 6 months, indicating persistent cognitive impairment. In the basal ganglia group, early VEEG changes reflected a “rhythmic imbalance” pattern, with mild reductions in α-wave power, compensatory increases in β-wave power, and slight elevations in slow-wave activity. These alterations demonstrated predictive value for cognitive function at 6 months. The brainstem group presented with “regional slow-wave activity”, predominantly localized to posterior head regions. This pattern was associated with fluctuations in alertness and increased risk of attention deficits during the chronic phase.

ROC analysis in this study demonstrated that a composite predictive model combining P300 ERP latency and amplitude with quantitative VEEG spectral parameters such as α/β power ratio and enhanced θ-wave activity, which was measured at 7 days post-stroke, significantly predicted MMSE scores at 6 months across different lesion locations, including the frontal lobe, temporal lobe, and basal ganglia. Previous studies have highlighted the utility of P300 in forecasting cognitive impairment [34], whereas increased slow-wave activity on EEG reflects early neural plasticity deficits and diminished cognitive reserve after stroke [38, 39]. Our findings further confirmed that integrating P300 and VEEG parameters provided a more precise assessment of PSCI risk than did single indicators alone. This approach offers a valuable electrophysiological basis for early clinical intervention and tailored rehabilitation strategies. This finding was consistent with studies showing that multimodal models outperform single-modality predictors in discrimination and calibration, with meaningful net reclassification gains. Integrating electrophysiology with structural or perfusion imaging may further enhance lesion-specific risk mapping and clinical deployment.

However, this study had several limitations. First, it is a single-center study with a modest sample, so generalizability requires confirmation in larger multicenter cohorts. Second, cognition was mainly assessed with the MMSE, which is less sensitive for executive, attention, and visuospatial domains; adding the MoCA may better detect mild PSCI and improve model performance. Future work should adopt harmonized cognitive batteries (e.g., MoCA plus domain-specific tests) and pre-registered assessment windows to reduce heterogeneity. Third, inclusion windows by days from stroke onset were not prespecified and vascular subtypes were not determined, potentially increasing heterogeneity and diluting subtype-specific differences. Future studies will standardize imaging-based subtype classification and assessment windows to enhance validity.

## 5. Conclusions

In conclusion, P300 combined with VEEG demonstrated strong potential for early PSCI prediction, with the highest efficacy in frontal- and temporal-lobe stroke patients. This method offers a practical, objective means of stratifying PSCI risk based on lesion location, facilitating timely and targeted cognitive rehabilitation.

## Data Availability

The original contributions presented in the study are included in the article/**Supplementary Material**, further inquiries can be directed to the corresponding authors.

## Abbreviations

AUC, Area under the curve; BMI, Body mass index; DAR, Delta/alpha ratio; EEG, Electroencephalography; ERP, Event-related potential; PSCI, Post-stroke cognitive impairment; qEEG, quantitative EEG; ROC, Receiver operating characteristic; SD, Standard deviation; VEEG, video EEG.

## Supplementary Material

Supplementary material associated with this article can be found, in the online version, at https://doi.org/10.31083/RN45402.
