# Supplementary material for: Evaluation of Cognitive Function in Stroke Patients With Lesions in Different Brain Regions Using P300 Event-Related Potentials Combined With Video EEG
Source: Rev Neurol. 2025 Dec 18;80(11):45402. doi: 10.31083/RN45402 (PMC12781230; doi:10.31083/RN45402)
Supplement: Supplementary file 1 [file 1576-6578-80-11-45402-s1.zip › Supplementary Table 1.docx]

| Characteristic | Observation  (n = 214) | Control  (n = 60) | Test | P value |
| --- | --- | --- | --- | --- |
| Age, years | 61.8 ± 5.4 | 62.1 ± 5.1 | Welch’s t-test | 0.72 |
| Age band, 50–59 / 60–70 | 92 / 122 | 26 / 34 | χ² test | 0.88 |
| Sex, male/female | 126 / 88 | 35 / 25 | χ² test | 0.74 |
| BMI, kg/m² | 25.8 ± 3.6 | 25.5 ± 3.4 | Welch’s t-test | 0.61 |
| Education, years | 12 [9–16] | 12 [10–16] | Mann–Whitney U | 0.69 |
| Education strata, low / mid / high | 64 / 96 / 54 | 18 / 28 / 14 | χ² test | 0.93 |
| MMSE total (screening) | 27.3 ± 1.5 | 28.6 ± 0.8 | Welch’s t-test | <0.001 |
| Hypertension, n (%) | 118 (55.1%) | 31 (51.7%) | χ² or Fisher’s exact | 0.62 |
| Diabetes, n (%) | 46 (21.5%) | 11 (18.3%) | χ² or Fisher’s exact | 0.57 |
| Hyperlipidemia, n (%) | 102 (47.7%) | 27 (45.0%) | χ² or Fisher’s exact | 0.69 |
| Smoking, current, n (%) | 58 (27.1%) | 15 (25.0%) | χ² or Fisher’s exact | 0.73 |

**Supplementary Table 1. Baseline characteristics.**
